# Thermal Behavior of Biaxial Piezoelectric MEMS-Scanners

**DOI:** 10.3390/s23239538

**Published:** 2023-11-30

**Authors:** Laurent Mollard, Christel Dieppedale, Antoine Hamelin, Gwenael Le Rhun, Jean Hue, Laurent Frey, Gael Castellan

**Affiliations:** University Grenoble Alpes, CEA, Leti, F-38000 Grenoble, France; christel.dieppedale@cea.fr (C.D.); antoine.hamelin@cea.fr (A.H.); gwenael.le-rhun@cea.fr (G.L.R.); jean.hue@cea.fr (J.H.); laurent.frey@cea.fr (L.F.); gael.castellan@cea.fr (G.C.)

**Keywords:** thermal behavior, 2D MEMS mirror, piezoelectric, Bragg reflector, high optical power management

## Abstract

This paper presents the thermal behavior of non-resonant (quasi-static) piezoelectric biaxial MEMS scanners with Bragg reflectors. These scanners were developed for LIDAR (LIght Detection And Ranging) applications using a pulsed 1550 nm laser with an average power of 2 W. At this power, a standard metal (gold) reflector can overheat and be damaged. The Bragg reflector developed here has up to 24 times lower absorption than gold, which limits heating of the mirror. However, the use of such a reflector involves a technological process completely different from that used for gold and induces, for example, different final stresses on the mirror. In view of the high requirements for optical power, the behavior of this reflector in the event of an increase in temperature needs to be studied and compared with the results of previous studies using gold reflectors. This paper shows that the Bragg reflector remains functional as the temperature rises and undergoes no detrimental deformation even when heated to 200 °C. In addition, the 2D-projection model revealed a 5% variation in optical angle at temperatures up to 150 °C and stability of 2D scanning during one hour of continuous use at 150 °C. The results of this study demonstrate that a biaxial piezoelectric MEMS scanner equipped with Bragg reflector technology can reach a maximum temperature of 150 °C, which is of the same order of magnitude as can be reached by scanners with gold reflectors.

## 1. Introduction

For most applications, including pico-projection and automotive and biomedical applications, MEMS scanners are of great interest due to their small size, low cost and low power consumption compared to standard mechanical beam-scanning systems. Numerous publications present potential applications for MEMS scanners, as well as the related requirements [1,2]. There has been particular focus recently on the development of LIDAR systems for autonomous driving systems. For long-range applications (distances greater than 100 m), the MEMS-LIDAR approach, based on a MEMS-scanner and using an incident laser wavelength of 1550 nm, currently appears to be the best option [3,4]. The main reason for using a 1550 nm laser is that the maximum allowable exposure for human eyes is higher at this wavelength than at 905 nm.

The 2D MEMS-scanner was developed with PZT (lead zirconium titanate) piezoelectric actuators on 8-inch silicon wafers using VLSI (very large-scale Integration) technology. The scanner’s design is compact compared with electromagnetic scanners, which require a bulky magnet to be integrated into the packaging. In addition, the PZT actuator is low-voltage (<25 V) compared to other types, such as electrostatic actuators (>150 V). Using this actuator, we obtained a total optical angle close to 8°, with a maximum driving voltage of 20 V [5]. As will be discussed later, the optical angle can be improved by pushing the design-drawing rules. In addition to these advantages, PZT actuators offer fast response times and low power consumption.

Long-range LIDAR detection requires high incident power. Our specification is an average incident optical power, at 1550 nm, of 2 W on the 2 × 2 mm^2^ mirror, which corresponds to a power density of 5000 W/m^2^. With a pulse laser, this specification corresponds to a few kW of peak power.

**Figure 1:**
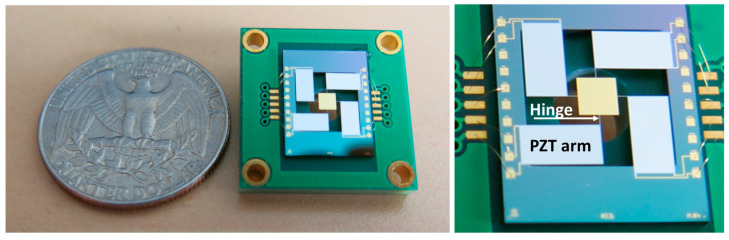
MEMS mirror, top view (**left**) and zoom on hinge and PZT arm (**right**).

Thus, the absorption (A) of the reflector is key to handling this significant incident optical power while also limiting heating of the mirror. Indeed, the power absorbed ${(P}_{a})$ inside the mirror is proportional to the reflector’s absorption (A): $P_{a}=A\times P_{i}$, where $P_{i}$ is the incident optical power. When the mirror is at thermal equilibrium, this absorbed optical power is equal to the power dissipated ${(P}_{d})$. Incident power can be dissipated in three ways: by surface radiation, by conduction through the arms or the air surrounding the scanner, or by convection. Free convection is often neglected because of the mirror’s dimensions, and dissipation is mainly attributed to conduction [6,7]. For our specific design, the main route of power dissipation was conduction through the arms. Air conduction is much lower because of the distances (of the order of 700 µm) between the mirror and the substrate. The thermal power dissipated by conduction ${(Q}_{cond})$ is proportional to the mirror’s temperature: $Q_{cond}=\frac{\left(T_{m}\;-\;T_{s}\right)}{R}$, where $T_{m}$ and $T_{s}$ are the temperature of the mirror and silicon substrate (considered at ambient temperature), respectively. R is the overall thermal resistance of the PZT arm ($R_{arm})$ and of the hinge ($R_{hinge})$, which are in series, as shown in Figure 1. This approximation leads to the following equations: $P_{a}≅Q_{cond}$ and $A\times R{\;\times \;P}_{i}\propto T_{m}-T_{s}$.

R can be expressed as follows: $R=R_{arm}+R_{hinge}=\frac{e_{arm}}{\lambda \;*\;S_{arm}}+\frac{e_{hinge}}{\lambda \;*\;S_{hinge}}$, with $R_{arm}=\frac{e_{arm}}{\lambda \;*\;S_{arm}}$ and $R_{hinge}=\frac{e_{hinge}}{\lambda \;*\;S_{hinge}}$. In this equation, $e_{arm/hinge}$ are the length of the arm and hinge, respectively, and $S_{arm/hinge}$ is the cross-section area (product of width and thickness) of the arm and hinge, respectively. The value of λ can be defined, initially, as the thermal conductivity of silicon in view of the very low thickness of the other layers. In the specific case of our mirror, with an optical angle of 8°, $R_{arm}$ is 15× lower than $R_{hinge}$, and consequently $R\approx R_{hinge}$.

Finally, the temperature of the mirror $T_{m}$ can be expressed as follows:

$$T_{m}-T_{s}\propto A\times R_{hinge}{\;\times \;P}_{i}$$

One way of increasing the optical angle in the near future will be to limit the stiffness of the hinge and therefore further reduce its width, which will result in a greater $R_{hinge}$. This increase in the value of $R_{hinge}$ will limit the power dissipated by conduction through the arms and therefore lead to an increase in mirror temperature. One way of limiting this temperature rise is to reduce the reflector’s absorption in order to reconcile greater angular deflection with limited mirror heating.

As a potential solution, a low-absorption Bragg reflector compatible with piezoelectric actuation was previously developed [5]. The optical absorption by this Bragg reflector was up to 24-fold lower than absorption by a standard gold reflector. This Bragg reflector consists of repeated (*n* times) dielectric bilayers of amorphous silicon (a-Si, 110 nm) and oxide (SiO_2_, 305 nm).

However, the impact of increased temperature on scanner performance needs to be assessed in the context of high optical power or high-temperature environments. The impact of increasing scanner temperature on the performance and characteristics (flatness, absorption, laser-induced damage threshold, etc.) has been studied elsewhere [6,7,8,9]. However, only technological processes using metal reflectors have been reported. Because the technological process with the Bragg reflector is different, the final stress of the multilayers will also be different, as will the way they change with increasing temperature. Their performance as the temperature rises must be investigated.

The main aims of the study are to ensure that the mirror remains functional even when the temperature rises, to study the deformations a rise in temperature could induce and to determine the maximum operating temperature for this Bragg scanner. The results will enable us not only to extend the use of such a mirror to a high-temperature environment, but also to consider applications other than the LIDAR that might require higher optical power.

## 2. Results and Discussion

The Bragg reflector was manufactured by physical vapor deposition (PVD) to avoid degradation of the PZT performance. It consists of several (*n*) bilayers of amorphous silicon (110 nm) and oxide (305 nm), depending on the target reflectivity and absorption. A two-bilayer (*n* = 2) Bragg reflector was chosen for the LIDAR application.

In the PZT process, process used was the most efficient in terms of material thickness, annealing and electrodes [10]. The 535 nm PZT piezoelectric film was deposited by sol-gel chemical solution deposition (CSD) on 8-inch silicon wafers. This film consists of 10 layers of a commercial PZT (52/48) solution from Mitsubishi Materials Corporation. Each layer of PZT was spun, dried at 130 °C and calcinated at 360 °C. Crystallization and densification annealing were performed at 700 °C under oxygen for 1 min using a Jipelec RTA (Rapid Thermal Annealing) furnace from Annealsys (Fr.). A first round of annealing was done on the first layer after it was coated to promote the desired (100) orientation. Subsequently, annealing was performed after coating of every three layers. This standard process does not include a hot-poling step after the deposition of PZT.

**Figure 2:**
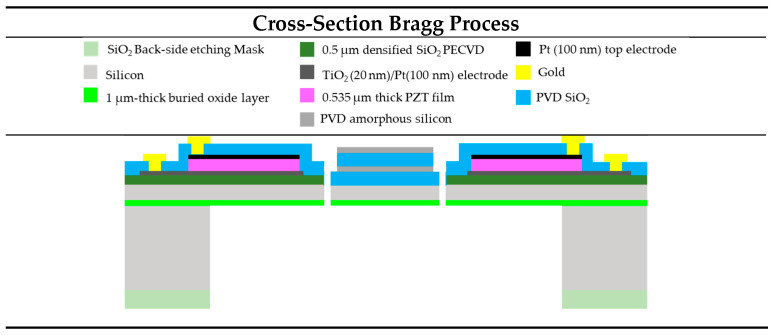
Cross-section of scanner with Bragg reflector.

Figure 2 shows a schematic cross-section view of our scanner. The fabrication process flow and PZT characterizations have been previously reported [5].

To create the Bragg bilayers, an Endura PVD magnetron sputtering chamber from Applied Material (U.S.A.) was used, having been loaded with a silicon target for the 110 nm amorphous silicon layers. The 305 nm silicon dioxide layers were deposited in the same chamber by reactive sputtering of DC-generated plasma.

The evolution of the mirror’s flatness and of the 2D-scan patterns with increasing temperature were experimentally characterized. These characterizations were carried out with the scanner thermalized on a hot plate. This experimental protocol has two principal advantages: it allows precise control of the scanner temperature, and it is compatible with a wide range of temperatures (from ambient to 200 °C).

### 2.1. Mirror Planarity with Temperature

Due to residual stresses in the multiple layers making up the mirror, the mirror was not perfectly flat. A concave or convex deformation of the mirror is often observed after fabrication, and this deformation affects beam reflection. The deformation can be minimized through technological means, for example, by changing the thickness and stress of the layers on the top and bottom of the mirror. The deposition process was first optimized to minimize internal multilayer stress. The average internal stress of the Bragg reflector bilayers (*n* = 2) was measured at −155 MPa on a complete wafer after deposition. This low compressive stress makes it possible to minimize mirror deformation induced by stress.

**Figure 3:**
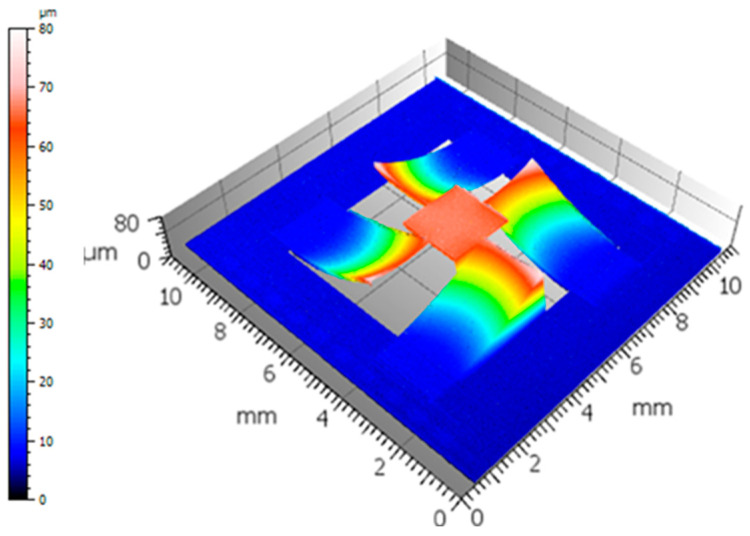
Scanner deformation of a Bragg reflector (*n* = 2) along the Z-axis near ambient temperature (30 °C).

At the same time, the scanner’s Z-deformation was measured after the Bragg process, using an Altisurf 520 tool from Altimet (Fr.). Figure 3 shows an overview of the scanner’s deformation along the Z axis at ambient temperature.

The mirror clearly displayed a convex deformation at 30 °C. The static radius of curvature (SRC), measured along the diagonal of the mirror over 2 mm and centered on the mirror, was estimated to be 230 mm. The 4 mm-long actuators had a positive deflection of 57 µm, which was induced by compressive stress affecting the whole multilayer stack.

**Figure 4:**
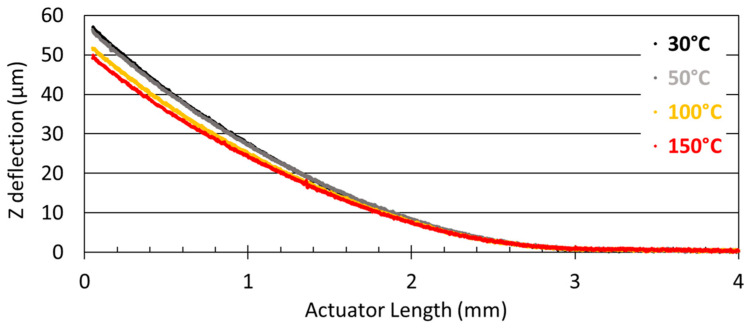
Z deflection of the PZT arms decreases with increasing temperature.

The evolution of Z deflection by the actuators and the mirror, as a function of temperature, was characterized over the temperature range from 30 °C to 200 °C. To carry out this evaluation, the scanner was thermalized on the heating plate integrated into the Altisurf 520 tool. As shown in Figure 4, increasing scanner temperature resulted in a 10% decrease in the Z-deflection, which reached a value close to 50 µm at 150 °C.

This expected evolution is consistent with published analytical models of multilayers [11,12]. The thermal stress $\left(\sigma_{Ti}\right)$ between a film (i) and a substrate (s) is defined by the equation

$$\sigma_{Ti}={E_{i}}^{\prime }\left(\alpha_{i}-\alpha_{s}\right)\left(T_{dep}-T_{sub}\right),$$

where $\alpha_{i,S}$ are the thermal expansion coefficients of the film (i) and substrate (s),$\;E_{i,S}^{\prime }=\frac{E_{i,S}}{\left(1\;-\;\vartheta_{i,S}\right)}$ corresponds to their biaxial Young modulus, $\vartheta_{i,S}$ is the Poisson ratio and $T_{dep}$ and $T_{sub}$ are, respectively, the temperatures of deposition and operation.

Taking the values of ${E_{i,s}}^{\prime }$ and $\alpha_{i,s}$ reported in [8] and our $T_{dep}$ values, as shown in Table 1, the formula clearly shows that increasing the operating temperature, $T_{sub}$, from 30 °C to 200 °C induces a decrease in overall stress, which in turn reduces beam deflection.

The radius of curvature of a multilayer beam can be estimated using the following formula [11]:

$$\frac{1}{r}=\sum_{i=1}^{n}\frac{6{E_{i}}^{\prime }t_{i}(\alpha_{i}-\alpha_{S})∆T}{{E_{s}}^{\prime }{t_{S}}^{2}}$$

where $t_{i,S}$ the thickness of the layer (i) or substrate (s). This last equation is a first-order approximation that omits terms with orders higher than $t_{i}$. This omission is consistent with the $t_{i}$ layer thicknesses being at least 20-fold thinner than the substrate thickness. However, these models do not account for any bending moment on the beams. This assumption does not apply in our case because the beams are connected to the mirror. Nevertheless, the model allowed us to determine that the largest ${E_{i}}^{\prime }t_{i}$ contributions were related to the bottom 1 µm-SiO_2_ film, which was present under the substrate. To a lesser extent, the PZT layer contributed as well. Some layers, like TiO_2_, may not be taken into account because of their negligible thickness. As a result, the overall radius of curvature of the actuators, and the degree of deflection, is mainly to the result of the compressive 1 µm-bottom SiO_2_ film present under the mirror. It should be noted that the initial deformation of the actuators had no direct impact on the optical angle attained; it affected only the Z-position of the mirror.

**Figure 5:**
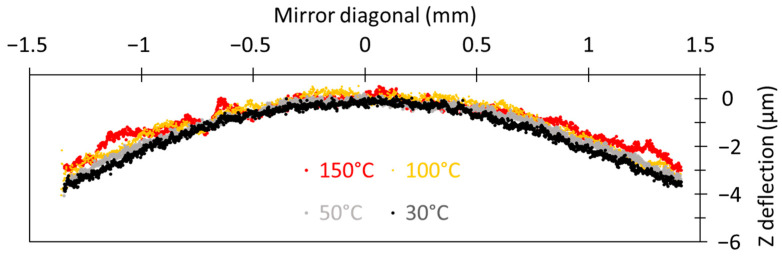
Bragg (*n* = 2) mirror planarity at different temperatures. The mirror was scanned along its diagonal.

Figure 5 shows changes to the mirror’s flatness as a function of temperature. The multilayer stack on the mirror is different from that present on the actuator. The mirror tends to flatten as the temperature increases. The increase in Z-signal noise with increasing temperature is linked to the measurement tool and does not reflect a change in the reflector surface, as the reflector was deposited at a higher temperature (250 °C).

Despite this noise, from experimental measurements, the SRC was estimated to be close to 340 mm at 150 °C. In future developments, this mirror curvature will need to be reduced to achieve the Rayleigh criteria: a Z-deformation of less than λ/4. This result could be achieved by, for example, increasing the thickness of the SiO_2_ layer present under the mirror or by adding a tensile layer on top of the silicon substrate.

### 2.2. 2D Projected Scan with Temperature

A key parameter to characterize is how the 2D scan of the reflected beam evolves as a function of scanner temperature. This measurement is used to determine and extrapolate the potential variation in the total optical angle depending on the mirror’s temperature in the context of a LIDAR application. The X and Y coordinates of the optical angle determine the size of the scene that the LIDAR can image.

The scanner was thermalized at temperatures between 30 °C and 150 °C, and the optical angles were measured using a class-2 visible laser reflected by the mirror and projected onto a screen. A camera was used to track the position of the laser on the screen by continuously measuring its barycenter. If the optical path (l) (around 130 mm) between the screen and the scanner and the position $\left(x,y\right)$ of the barycenter of the laser on the screen are known, the X and Y $\left(\theta_{x},\theta_{y}\right)$ coordinates of the optical angle of the spot can be deduced using tan $\left(\theta_{x\;or\;y}\right)$ = $\frac{x\;or\;y}{l}$. The absolute uncertainty of the optical angle, which is linked to how precisely the optical path is measured, was estimated to be 0.2° on our test bench. The scanner temperature was limited to 150 °C because electrical breakdowns are often observed when our technological process is used at temperatures exceeding 200 °C.

**Figure 6:**
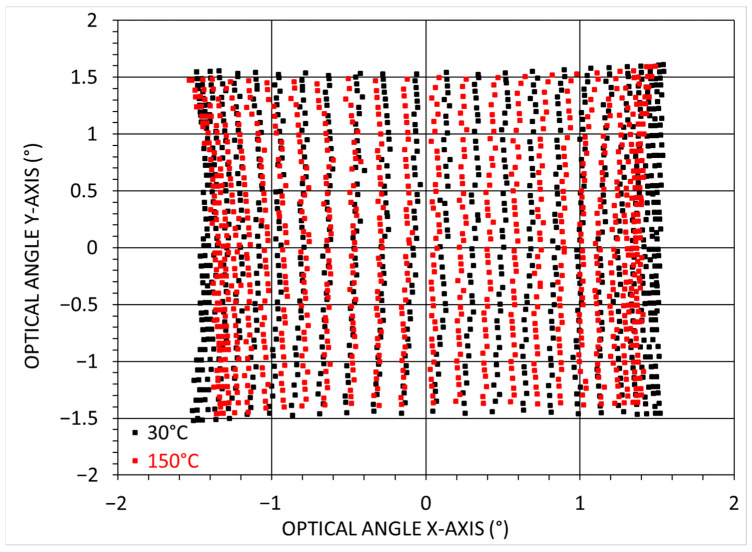
2D-scanning representation at 30 °C (black) and 150 °C (red).

A 2D scan was performed with a 200 Hz sinusoidal driving signal (X-axis) for the fast axis and a 4 Hz ramp for the slow axis (Y-axis). The PZT supply voltage was swept from 0 V to a maximum of 20 V with $V_{AC}=V_{DC}=10\;V$. The total optical angle $\theta_{x}$ and $\theta_{y}$ was measured at y = 0 and x = 0, respectively. In addition, for each of the four temperatures tested, the total duration of the measurement was at least 30 min, including thermalization and the time required for 2D scanning. Figure 6 shows the optical angles measured for one of our scanners at 30 °C and 150 °C. A slight decrease can be observed in both axes of the scanning pattern. Additionally, it is worth noting a minor distortion in the 2D scan at 150 °C. This distortion will require further examination.

**Figure 7:**
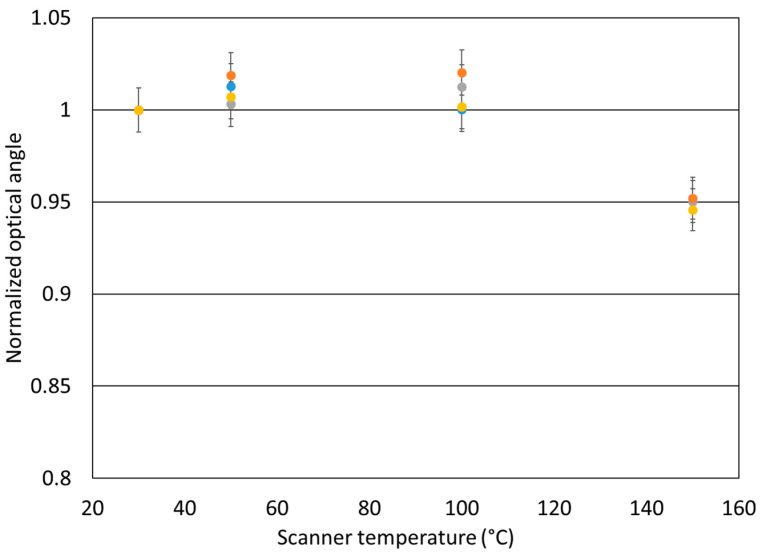
Optical angle $\theta_{e}$ (normalized relative to angle measured at 30 °C) as a function of scanner temperature done with four identical mirrors.

Four identical Bragg mirrors were used for this test, as shown in Figure 7. These mirrors have exactly the same design, the same Bragg reflector and the same technological process flow. The evolution of the effective optical angle, $\theta_{e}=\sqrt{\theta_{x}\theta_{y}}$, as a function of changes in scanner temperature from 30 °C to 150 °C is reported. The changes observed correlated well with previously published results [8,13,14,15].

Mirror deflection thus results from the movement of the PZT actuators and, consequently, of the effective transverse piezoelectric coefficient $e_{31,f}$, based on the inverse piezoelectric effect. Indeed, the piezoelectric stress ($\sigma_{P})$ can be expressed as $\sigma_{P}=-e_{31,f}E_{z}$ [13], where $E_{z}$ is the transverse electric field. In addition, $e_{31,f}$ is proportional to $P_{S}$, the saturation polarization, and $\varepsilon_{33}$, the relative permittivity measured out of plane, as $e_{31,f}\propto -2\varepsilon_{33}P_{3}$. This coefficient is highly dependent on the operating temperature of the mirror. Theoretical calculations [14] predict that $e_{31,f}$ will increase with temperature. In contrast, other authors [8,15] have reported experimental results showing that $e_{31,f}$ increases to a maximum (near 40 °C) and then gradually decreases at higher temperatures (up to 200 °C).

After deposition, ferroelectrics like PZT may not be polarized, in which case they will have poor piezoelectric properties. Poling treatment, which involves applying a voltage well above the coercive voltage, orients the piezoelectric domains in the same direction. However, after poling, the piezoelectric coefficient slowly decays due to rearrangement of the domains towards their equilibrium state. If poling is performed at a high temperature (hot poling), the mobility of the ferroelectric domains increases significantly, allowing a better alignment in the direction of the poling field [10]. Nevertheless, it should be noted that this increase in $e_{31,f}$ after hot poling has been observed only at ambient temperature. Another study [15] with an initial poling step of 25 V at ambient temperature and intermediate poling steps at higher temperatures found that $e_{31,f}$ decreases with increasing temperature. The two curves converged at high temperatures (near 200 °C).

This case is different, as the temperature of the scanner increases progressively with continuous polarization of the PZT. A similar case has been reported elsewhere [8]. Dahl-Hansen et al. followed the deflection tilt of a unipolar micro-mirror driven by a 20 V signal at a frequency of 1.5 kHz while varying the mirror temperature stepwise from ambient temperature to 175 °C. Our study shows a similar trend, with an increase in the optical angle between 50 °C and 100 °C and a decrease at 150 °C. The value of the maximum is more difficult to determine given the uncertainty of the measurements. In addition, one of our samples underwent an electrical breakdown at 100 °C, reflecting degradation at high temperature. This decrease in optical angle with increasing temperature has been attributed mainly to the decrease in $P_{s}$ as the scanner temperature approaches the Curie temperature (near 350 °C in our case) [8,16,17]. Alteration of film stress is also reported to have a potential impact [8] but is considered to contribute only slightly to the evolution of $e_{31,f}$ [17].

The decrease in optical angle observed in Figure 7 was close to 5%, which is consistent with values reported elsewhere [8]. However, this evolution of the optical angle, which is linked to the actuation power, even if it is limited to a change of few percent, must be taken into account in the context of future use within a LIDAR system. This value has direct implications for the global area scanned from the scene. Limiting the temperature increase by means of a low-absorption reflector is a first response to this issue, but that solution could also be coupled to others, like those based on the influence of PZT poling or hot-poling conditions, as has been reported [9]. These and other studies, such as investigations focusing on the evolution of $P_{s}$, should be explored further in the future.

**Figure 8:**
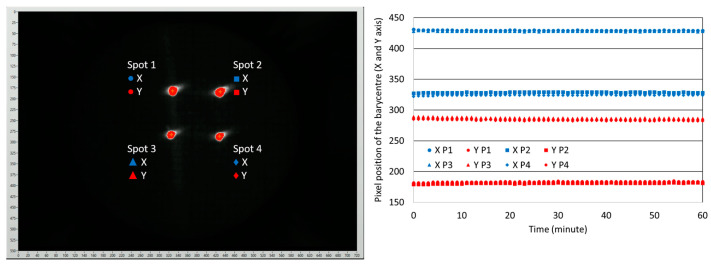
Projected image of the four spots on the screen (**left**) and shift in the barycenter (X and Y) of the four spots (**right**).

The last parameter checked was the stability of the 2D scan at an operating temperature of 150 °C. To conduct this investigation, an image of four spots, as shown in Figure 8, was continuously projected onto the screen for one hour. An image of the screen was taken every minute by a 720-by-540-pixel camera (X and Y) and the barycenter of each of the spots was calculated. The shift in the X and Y positions of the barycenter, is reported in pixels.

The standard deviation $S_{n-1}$ of the barycenter positions of the four spots, calculated in pixels for both X and Y axis, was estimated using the following formula: $S_{n-1}=\sqrt{\frac{\sum (x\;-\;\bar{x})2}{n\;-\;1}}$, where x is the value for one sample, $\bar{x}$ is the mean value of all the samples and *n* is the number of samples. In our case, *n* = 60. The maximum value of $S_{n-1}$ is close to 1 pixel. This value is the smallest deviation that can be measured using our test bench. Thus, in our case, the relative deviation of the optical angle was close to 0.03°.

In conclusion, no significant deviation of the optical angle at a scanner temperature of 150 °C was observed for continuous one-hour measurements, demonstrating the stable performance of the PZT actuator.

Further studies are needed to verify that PZT performance is not affected by a return to ambient temperature after operation at different temperatures. The potential impact of a hot-poling step remains to be investigated as well.

## 3. Conclusions

In this paper, we report on the thermal behavior of a biaxial piezoelectric MEMS-scanner with integrated Bragg-reflector technology. We first demonstrate that the flatness of the mirror and actuators compensates for increases in internal stresses in the multilayer stack with increasing temperature. Better adaptation of the thickness of the layers making up the mirror could further improve initial planarity to provide better optical quality of the reflected beam. We then looked at the evolution of the 2D projected pattern with increasing scanner temperature. An optical angle variation close to 5% was observed at 150 °C, which is consistent with previously reported values. Such results could be taken into account in the case of application at elevated temperature to adapt the FoV of the scanner. Finally, no significant deviation of the optical angle was observed for continuous measurements lasting one hour at a scanner temperature of 150 °C. The results presented here demonstrate that a biaxial piezoelectric MEMS scanner equipped with Bragg reflector technology can reach a maximum operating temperature near 150 °C, which is of the same order of magnitude as scanners equipped with a gold reflector. Moreover, thanks to its low-absorption Bragg reflector, this technology will make it possible in the future to increase angular deflection by reducing the width of the mirror hinge while limiting mirror heating. This technology thus paves the way for applications requiring high optical power or high-temperature environments. Finally, now that the maximum operating temperature is known, future simulation models will be able to estimate the maximum permissible incident power using the design of the scanner and the absorption of the Bragg reflector.

## Figures and Tables

**Table 1:** Values of material characteristics from [8] and our actuator technology process.

Material	E_i,s_′ [GPa] [8]	α_i,s_(10^−6^) [8]	*t_i,s_* (µm)	*T_dep_* [ °C]
Pt	271	9	0.1	450
PZT	119	6	0.55	700
Pt	271	9	0.1	450
SiO_2_	100	0.6	0.5	250
Si	236	4.3 (30 °C) 5.5 (150 °C)	20	N/A
SiO_2_	100	0.6	1	1050 [8]

## Data Availability

Data are contained within the article.
